# Implementing a Digital HIV Care Navigation Intervention (Health eNav): Protocol for a Feasibility Study

**DOI:** 10.2196/16406

**Published:** 2019-11-08

**Authors:** Sean Arayasirikul, Dillon Trujillo, Caitlin M Turner, Victory Le, Erin C Wilson

**Affiliations:** 1 Department of Pediatrics University of California San Francisco San Francisco, CA United States; 2 Department of Psychiatry University of California San Francisco San Francisco, CA United States; 3 Center for Public Health Research San Francisco Department of Public Health San Francisco, CA United States

**Keywords:** HIV/AIDS, digital navigation, young people living with HIV, mHealth

## Abstract

**Background:**

Young racial and ethnic minority men who have sex with men (MSM) and trans women are disproportionately affected by HIV and AIDS in the United States. Unrecognized infection, due to a low uptake of HIV testing, and poor linkage to care are driving forces of ongoing HIV transmission among young racial and ethnic minority MSM and trans women. Internet and mobile technologies, in combination with social network-based approaches, offer great potential to overcome and address barriers to care and effectively disseminate interventions.

**Objective:**

We describe Health eNavigation (Health eNav), a digital HIV care navigation intervention that extends supportive care structures beyond clinic walls to serve youth and young adults living with HIV who are newly diagnosed, not linked to care, out of care, and not virally suppressed, at times when they need support the most.

**Methods:**

This study leverages ecological momentary assessments for a period of 90 days and uses person-delivered short message service text messages to provide participants with digital HIV care navigation over a 6-month period. We aim to improve engagement, linkage, and retention in HIV care and improve viral suppression. Digital HIV care navigation includes the following components: (1) HIV care navigation, (2) health promotion, (3) motivational interviewing, and (4) digital social support.

**Results:**

Recruitment began on November 18, 2016; enrollment closed on May 31, 2018. Intervention delivery ended on November 30, 2018, and follow-up evaluations concluded on October 31, 2019. In this paper, we present baseline sample characteristics.

**Conclusions:**

We discuss real-world strategies and challenges in delivering the digital HIV care navigation intervention in a city-level, public health setting.

**International Registered Report Identifier (IRRID):**

DERR1-10.2196/16406

## Introduction

### Overview

Young racial and ethnic minority men who have sex with men (MSM) and trans women are disproportionately affected by HIV and AIDS in the United States [1-5]. Although MSM represent just 2% of the US population, this group accounts for 57% of all new HIV infections and is the only risk group with new HIV infections rising each year [6]. The US Centers for Disease Control and Prevention estimates that among all MSM in the United States, black and Latino MSM account for the majority of HIV infections [7]. A recent study found an HIV incidence among black MSM to be 4.16% per year; in a simulated cohort, authors found that almost 40% of black MSM would be infected with HIV by age 30 and approximately 60% by age 40 [8]. While there has been progress in reducing infections among racial minorities, new HIV infections among black MSM increased 22%, with the largest increase among young men who have sex with men (YMSM) [7]. Nationally, almost two-thirds of new infections in 2008 occurred among those 13-29 years of age, most of which were among African American and Latino MSM [6]. Similar to young racial and ethnic minority MSM, young racial and ethnic minority trans women also bear a huge burden of HIV infections in the United States [9,10]. Data in San Francisco, California, found a 39.5% HIV prevalence among adult trans women [11] and a 7% HIV prevalence among young trans women [12].

Unrecognized infection, due to a low uptake of HIV testing, and poor linkage to care are driving forces of ongoing HIV transmission among young racial and ethnic minority MSM and trans women. Compared to older MSM and white MSM, YMSM (aged 18-29 years) (63%) and racial and ethnic minority MSM (54%) were more likely to be unaware of their HIV infection [10]. In terms of linkage to care, 62% of African Americans and 67% of Latinos were linked to care within 3 months of diagnosis compared to 71% of whites; only 56% of those between 25 and 34 years of age were linked to care compared to about 70% in other age categories [10]. Ultimately, these subgroups were less likely to achieve viral suppression. Similarly, the burden of HIV in trans women is exacerbated by unrecognized infections and low access to HIV care [10]. In 2012, counseling and testing data from San Francisco public testing sites found that of the 17,898 HIV tests over the year, only 403 HIV tests were conducted with trans women [13]. Findings from a study of adult trans women living with HIV in the San Francisco Bay Area found that 77% of participants were linked to care but only 44% were virologically suppressed [14]. Research has found that trans women with HIV in San Francisco have a significantly higher average aggregate viral load (ie, *community viral load*) compared to other populations [15].

Internet and mobile technologies, in combination with social network-based approaches, offer great potential to overcome and address barriers to care and effectively disseminate interventions. Traditional delivery methods of linkage and engagement services for HIV care services may be less effective at reaching at-risk youth and young adult MSM and trans women living with HIV in today’s social media-driven environment. As more individuals now have access to the Internet and other mobile technologies, social networking online and seeking health-related information on the Internet has become increasingly popular, especially among young sexual and gender minorities. Recent innovations in online methods for increasing HIV testing, initiating partner interventions and behavioral interventions, HIV care, self-management, and provider care have also demonstrated efficacy comparable to face-to-face interventions [16,17]. Interventions that leverage mobile technology and social media have also been found to have a greater impact in influencing behavior than radio and television campaigns [16]. There is evidence that social media can be effectively utilized to connect young adults with HIV and sexually transmitted disease information and increase condom use [18]. This is particularly relevant to the sociocultural contexts of young racial and ethnic minority MSM and trans women who experience homophobia and transphobia, both within their own racial and ethnic communities and the larger society; this makes them often more hidden and inaccessible through traditional public health outreach efforts [9,19]. Through accessing and receiving Internet- and/or mobile technology-based HIV interventions, these young racial and sexual minority individuals can remain safe and maintain their privacy. Additionally, since youth and young adults have large social networks online [20], interventions delivered on the Internet or through mobile technology may have greater diffusion effects. Social network members can provide both tangible and intangible resources or support, which may facilitate or protect against heath-related risks [21]. Furthermore, social network-specific norms can affect individuals’ attitudes regarding sex, risk behaviors, and health-seeking behaviors [22].

Mobile-based interventions have high promise for engaging youth and young adults in their HIV care. Mobile phones represent a common thread for communication among almost all youth and young adults in the United States, where approximately 95% of those aged 18-29 years own their own mobile phone [23]. Importantly, according to the 2012 National Health Interview Survey, more than 70% of those living in wireless-only households (ie, with no landline) in 2011 were at or below 200% of the federal poverty threshold, contradicting the conventionally held idea that use of mobile technology is concentrated among better-resourced people [24]. In fact, mobile technology today is used by almost all Americans in all socioeconomic groups and by higher percentages of African Americans and Latinos than whites [24]. Short message service (SMS) text messaging via mobile phones has been used to provide sexual health information to young people [25]. A South African study used SMS text messaging for all participant interactions, from recruitment through to final follow-up, and found that 10 motivational-style SMS text messages increased HIV testing rates to a statistically significant degree when compared to the control group [26]. Furthermore, the WelTel Kenya1 trial demonstrated that SMS text messaging support via weekly messages to participants improved adherence to HIV treatment medications or antiretroviral therapy (ART) and increased viral load suppression; this occurred when participants were required to respond regarding whether they were doing well or if there was a problem [27]. SMS text messaging alerts are also relatively unobtrusive, offering the user confidentiality in environments where HIV is often taboo.

Issues that youth and young adults living with HIV experience are complex and may benefit from a combination of digital and mobile health technologies and clinical and community-based interventions to achieve positive health outcomes. Traditional HIV care services must move outside of clinical settings to incorporate digital and social media technologies. The San Francisco Department of Public Health’s Center for Public Health Research developed a digital HIV care navigation intervention called Health eNavigation (Health eNav), which is designed for young and young adult (ages 18-34) MSM and trans women living with HIV. Among youth and young adults, linkage to care is relatively high at more than 80%, but there is a steep drop-off in retention in care (ie, <50%) and low viral suppression (ie, <70%) 12 months from diagnosis [13]. Youth and young adults are a subpopulation in particular need of interventions to improve linkage, retention, and engagement in HIV care. Many youth and young adults may not have a medical home (ie, a consistent relationship with and access to a primary care provider) due to population-specific challenges and barriers, such as homelessness, needs related to identity development, and job insecurity.

### Intervention Description

#### Overview

Health eNav is a 6-month, digital HIV care navigation intervention leveraging SMS text messaging to provide digital HIV care navigation services to young people living with HIV. During the intervention, participants are connected to their own digital HIV care navigator and receive the following components: (1) digital HIV care navigation and (2) ecological momentary assessments. Through the use of technology, Health eNav extends supportive care structures beyond clinic walls at times when youth and young adults living with HIV who are newly diagnosed, not linked to care, out of care, and not virally suppressed need support the most. Health eNav is an engagement and retention-in-HIV-care intervention, aimed at addressing critical gaps and barriers to successfully identifying, linking, engaging, and retaining youth and young adults living with HIV in medical care. The goal of Health eNav is to improve outcomes across the HIV care continuum, specifically retention in HIV care, ART initiation, and viral suppression.

Health eNav seeks to improve health outcomes by amplifying the reach and value of the patient-centered medical home (PCMH) model [28] with the use of digital technology. This PCMH model uses a care team approach to provide patients with focused and culturally relevant services, strong provider-patient relationships, the elimination of barriers to care, and increased efficiency and quality of care. In addition to the PCMH model, Health eNav delivers digital HIV care navigation that aligns with the chronic care model [29]. Health eNav seeks to provide increased linkages to community resources in a community-driven, cost-effective way; promote self-management that empowers participants to take an active role in their health; and offer clinical decision support, information sharing, and proactive care in real time [29,30].

#### Digital HIV Care Navigation

Digital HIV care navigation includes the following: (1) HIV care navigation, (2) health promotion, (3) motivational interviewing, and (4) digital social support.

#### HIV Care Navigation

HIV care navigation guides participants in knowing where, when, and how to access all health and related services and increases access to appropriate resources [31]. HIV care navigation services include the coordination of, and/or referrals to, the following services: (1) primary medical care, (2) specialty care, (3) mental health care and substance abuse services, (4) imaging and other diagnostic services, (5) laboratory services, (6) health insurance, (7) housing, and (8) benefits, entitlements, and public assistance.

#### Health Promotion and Education

Health promotion and education ensures optimal health literacy for all participants by providing information on the biology of HIV, disease management, communication with providers, risk reduction and healthy behavior, and ART adherence. Health promotion content is tailored, personalized, and specific to the needs of each participant; it is also documented in their individual care plan and updated on an ongoing basis. Health promotion and education are delivered to meet participants’ educational, developmental, language, gender, sexual, and cultural needs.

#### Motivational Interviewing

Motivational interviewing is a technique and a style of counseling that can help resolve the ambivalence that prevents patients from realizing their personal goals. Motivational interviewing builds on Carl Rogers' optimistic and humanistic theories about people's capabilities for exercising free choice and changing through a process of self-actualization. The therapeutic relationship for both Rogerian and motivational interviewers is a democratic partnership. Motivational interviewing is directive and aims at eliciting self-motivational statements and behavioral change from the client in addition to creating client discrepancy to enhance motivation for positive change [32,33]. Motivational interviewing activates the capability for beneficial change that everyone possesses [34]. Although some people can continue to change on their own, others require more formal treatment and support over the long journey of recovery. Even for participants with low readiness, motivational interviewing serves as a vital prelude to longer-term behavior change.

#### Social Support

The intervention provides patients with maximal access to social support from a digital navigator. The digital navigator maintains an open, nonjudgmental space with participants and provides social support through engaging in active listening, joint problem solving, and peer counseling on an as-needed and ongoing basis during the 6-month intervention period. They may also provide counseling to assist with disclosure where feasible and/or facilitate referrals to external social support providers (eg, community-based organizations) when appropriate.

#### Ecological Momentary Assessments and Harnessing the Power of Digital Sensing

Ubiquitous data collection in real time by using mobile technology can provide the critical contextual data needed to explain barriers to HIV care engagement. Ecological momentary assessment (EMA) is a behavioral medicine method used to collect data close in time to participants’ experience and in their natural environment, shedding light on the dynamics of behavior in the context of real-world settings [35-38]. EMAs are delivered to participants in the form of a short, daily, SMS text message survey to assess or sense early indicators of barriers and facilitators to HIV care engagement and treatment adherence. In Health eNav, EMAs gauge participants’ daily emotional affective state, mental health, substance use, and other risk behaviors known to directly affect HIV care engagement and treatment adherence. Furthermore, EMA data are fed back into digital HIV care navigation through a dashboard. Digital sensing of daily changes in affect and/or mental health by using EMAs can prompt tailored digital discussions, facilitate timely personalized referrals, and inform our understanding of early predictors of engagement in HIV care and treatment adherence.

## Methods

### Reporting and Design

We have followed the Standard Protocol Items: Recommendations for Interventional Trials guidelines for reporting on protocols [39]. This is a single-arm, prospective, explanatory, mixed-methods, pre-post design feasibility study.

### Setting

This intervention was housed physically in the Center for Public Health Research at the San Francisco Department of Public Health; however, most of the engagement in the intervention was virtual, through SMS text messaging.

### Participants

Eligible participants were youth and young adults, aged 18-34 years, diagnosed with HIV infection, who identify as an MSM or a trans woman and report living in San Francisco, California. Eligible participants also met at least one of the following criteria: (1) newly diagnosed with HIV or have tested HIV positive for the first time within the last 12 months prior to enrollment, (2) not linked to HIV medical care or are aware of their HIV infection status but have never engaged in care or never had an HIV medical visit after being diagnosed with HIV, (3) out of care or diagnosed with HIV more than 12 months prior to enrollment and had a gap in their HIV care that was longer than 6 months, within the last 24 months, and (4) not virally suppressed or have a viral load of at least 200 copies/mL at their last lab test. If participants did not have access to a mobile phone, they were provided with a mobile phone, 2 years of cellular service, and unlimited SMS text messaging.

### Ethics Approval

All procedures performed in studies involving human participants were in accordance with the ethical standards of the institutional and/or national research committee and with the 1964 Helsinki declaration and its later amendments or comparable ethical standards. This study protocol was approved by the Institutional Review Board (IRB) at the University of California, San Francisco (IRB number: 16-19675).

### Recruitment

Recruitment took place at San Francisco Department of Public Health community clinics and community-based organizations (CBOs). Convenience sampling was used to recruit potential participants from five clinics and CBOs in San Francisco serving young people living with HIV. Recruiting materials (eg, flyers) and presentations to staff were used to advertise study recruitment. Staff referred potential participants to the study through phone and email communication and/or in-person meetings. Enrolled participants were also invited to refer peers from their social network. We screened 171 potential participants and 140 were eligible; 20 of these individuals were lost to follow-up following screening. A total of 120 participants were enrolled into the study.

### Study Procedures and Data Collection Methods

**Figure 1:**
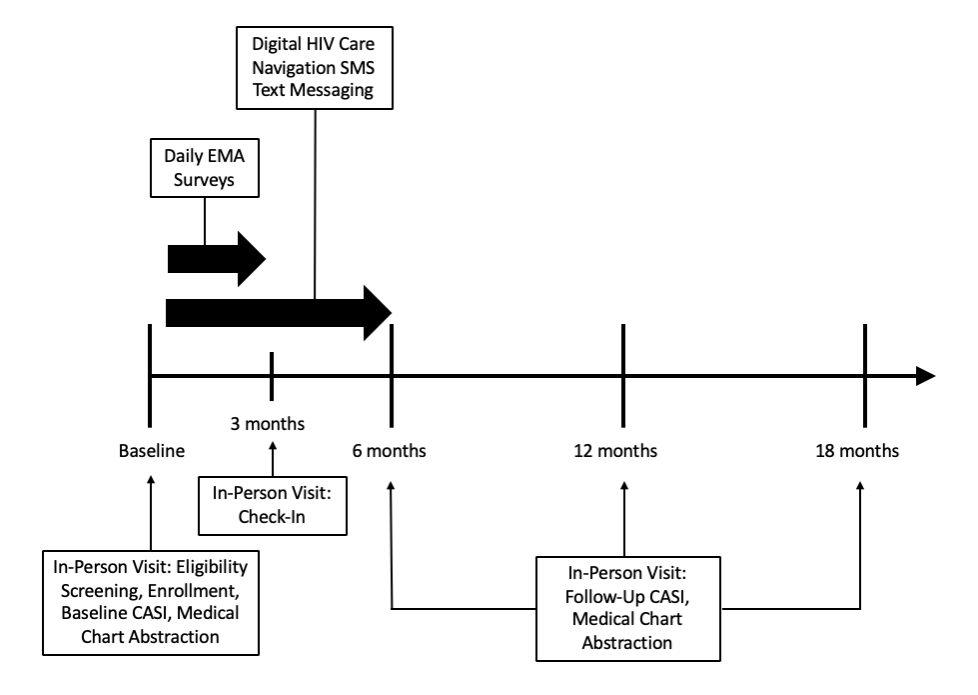
Health eNavigation (Health eNav) study design. CASI: computer-assisted self-interviewing; EMA: ecological momentary assessment; SMS: short message service.

At enrollment and baseline, participants were educated about the study, provided informed consent, and completed administrative paperwork. Participants met with their digital navigator and completed the following: a short qualitative interview, a comprehensive care plan, and computer-assisted self-interviewing (CASI) surveys. Participants received one-on-one training and instruction on SMS text messaging and completing EMA surveys. SMS text message and EMA data (ie, date and time, text, and responses) were collected using third-party vendors. Participants were sent automated EMA SMS text messages once per day at 8:00 am, 12:00 pm, or 8:00 pm for 90 consecutive days. They were required to respond to EMA surveys within 24 hours. Participants could receive between 17 and 31 daily EMA texts depending on their responses to programmed skip logic. EMAs tended to take less than 5 minutes to complete each day. Participants earned US $1 for each completed EMA survey; if participants completed 90% or more, they earned a bonus of US $100. At 3 months, participants met with their digital HIV care navigator in person for an informal check-in and were remunerated for the number of EMA surveys they completed during that period. Digital HIV care navigation continued for 6 months from enrollment. Participants were able to communicate with their digital HIV care navigator via SMS text message on an open schedule and conversations spanned any topic that participants would want discussed. The digital HIV care navigator would send weekly check-in messages to participants that included the following topics: general well-being, health education and health promotion, social support, and primary care appointment reminders, among others. CASIs were administered every 6 months for 18 months, and medical chart abstraction was conducted every 6 months for 18 months. Figure 1 describes the study design.

## Results

Recruitment began on November 18, 2016, and the first participant was enrolled on December 16, 2016; enrollment closed on May 31, 2018. Intervention delivery ended on November 30, 2018, and all study-related follow-up and procedures concluded on October 31, 2019. We present baseline sample characteristics in Table 1 below. Overall, the majority of participants were people of color, with the largest ethnic and racial minority subgroup being Latinx (38/120, 31.7%); 85.8% (103/120) identified as an MSM and 14.2% (17/120) identified as a gender minority. At baseline, many participants reported experiencing unstable housing, with only 32.5% of participants (39/120) renting or owning an apartment or home. Over one-third of participants (43/120, 35.8%) reported temporary or transitional housing, and 14.2% (17/120) reported living at a shelter or being homeless. Over half of the participants (68/120, 56.7%) reported having some college education or more. Nearly a third were newly diagnosed, and 6.7% (8/120) had never received primary HIV care. While the majority of participants have accessed HIV care within the last 6 months (99/120, 82.5%) and are currently on ART (92/120, 76.7%), only about half (65/120, 54.2%) were virally suppressed.

**Table 1:** Overall baseline sample characteristics for Health eNav^a^, 2017-2018 (N=120).

Characteristics			Value
**Demographics**			
	Age (years), mean (SD)		27.75 (4.07)
	**Race or ethnicity, n (%)**		
		Black or African American	22 (18.3)
		Hispanic or Latinx	38 (31.7)
		Other^b^ or multiple	28 (23.3)
		White	32 (26.7)
	**Gender identity, n (%)**		
		Male	103 (85.8)
		Trans woman, genderqueer, gender nonconforming, or other	17 (14.2)
**Socioeconomic factors, n (%)**			
	**Housing status**		
		Lives with a family member, friend, or partner who rents or owns a home	21 (17.5)
		Temporary or transitional housing^c^	43 (35.8)
		Homeless or lives in a shelter	17 (14.2)
		Rents or owns an apartment or house	39 (32.5)
	**Income in the last month (US $)**		
		0-250	30 (25.0)
		251-600	30 (25.0)
		601-1300	30 (25.0)
		1301 or more	29 (24.2)
	**Education**		
		High school or GED^d^	39 (32.5)
		Less than high school	13 (10.8)
		Some college or more	68 (56.7)
**HIV diagnosis status, n (%)**			
	Diagnosed in the last year		38 (31.7)
	Diagnosed more than 1 year ago		82 (68.3)
**Engagement in HIV care, n (%)**			
	**Received primary HIV care**		
		No	8 (6.7)
		Yes	112 (93.3)
	**Received primary HIV care in the last 6 months**		
		No	21 (17.5)
		Yes	99 (82.5)
	**Currently undertaking ART^e^**		
		No	27 (22.5)
		Yes	92 (76.7)
	**Last viral load test result**		
		Undetectable	65 (54.2)
		Detectable	32 (26.7)
		Unknown	15 (12.5)

^a^Health eNav: Health eNavigation.

^b^Includes participants who identified as American Indian or Alaska Native (n=6) or Asian (n=7).

^c^Includes participants who lived in single-room occupancy hotels, motels, boarding houses, halfway houses, drug treatment centers, independent living units, domestic violence shelters, battered persons' shelters, or *safe houses*.

^d^GED: General Educational Development.

^e^ART: antiretroviral therapy.

## Discussion

We were able to serve and provide digital HIV care navigation to 120 youth and young adults living with HIV in San Francisco. Some of our successes, lessons learned, and challenges and barriers are described.

### Successes

#### Delivering Personalized Social Support

The project was able to deliver personalized social support to youth and young adults living with HIV through innovative use of digital technology. For example, 1 participant was transitioning between HIV care providers and did not want his digital HIV care navigator to accompany him to the appointment. However, while he was in the waiting room, the participant was having a very difficult time fighting to not internalize the stigma from being in an HIV clinic. He went on to have a very difficult conversation with his new care provider. Meanwhile, he was able to speak with his personal digital HIV care navigator through this difficult clinical encounter. The digital HIV care navigator served as a caring source of support to listen to and support this participant; as a result, this participant was able to work through his feelings of stigma and other negative emotions to maintain linkage and engagement in care.

#### Collecting Real-Time Data

The project was able to collect timely individual-level data securely through mobile devices. After enrollment, participants received daily SMS text message surveys for 90 days on a variety of topics, including mental health and substance use. These data can impact individuals’ linkage, engagement, and retention in HIV care. They are reviewed by the HIV care navigator who can deliver personalized social support depending on how participants responded to text surveys. For example, 1 participant went approximately 30 days with no feelings of depression or anxiety. Suddenly, the participant started to indicate that they were feeling anxious or depressed. The digital HIV care navigator checked in with the participant via SMS text message and found out that they had not been able to successfully get a job, even after multiple interviews. The digital HIV care navigator was able to provide timely emotional and informational support to a participant that may not have shared this kind of information with their usual care provider.

### Challenges and Barriers

#### The Importance of Collaborative and Translational Communication

In our experience, technology vendors can be incredibly siloed. For example, few technology vendors have experience in health care or public health, let alone research. It is important to be acutely aware of these disciplinary boundaries as you may need to translate your research or public health needs for an audience who may not be aware of what exactly you do. Effective communication to foster understanding of purpose and organizational context will help aid negotiations and in scoping a project appropriately.

#### Keep Your Eye on the Front End and the Back End

It is important to emphasize both how the technology looks (ie, front end or user interface) and how it is structured in its programming code (ie, back end), as this can impact how data and metadata are collected and how databases are structured. For example, metadata are data that are collected on the back end. While the primary data might be SMS text messages, types of metadata might include date, time, and geolocation, among others. It may be important to consider how the data are collected, including metadata, and how the data are structured. For example, does the technology measure time using a 24-hour clock versus a 12-hour clock? Will the project need to calculate time? If so, the 24-hour clock might be a better measure to compute a new time variable using two time measurements. While seemingly minor, a detail like this might require a redesign with a hefty price tag, especially if there is a limited budget for adjustments.

#### Agree on a Contract That Lives On for the Length of a Project

When entering into a contract with a technology vendor, create a contract that spans the entire length of the project if possible. Important issues may include key software updates, ongoing technical support, and new features, among others. This will allow additional adjustments to be made along the way.

#### Tips for Implementation

1. Offer an alternative work schedule to incorporate flexibility in providing digital navigation and support conversations with participants at times that they prefer and are most accessible to them.
2. Prioritize initiating conversations with participants who have a higher acuity level versus those who have lower acuity.
3. Develop a quick reference guide of resources to provide to participants.
4. Utilize peers as digital HIV care navigators.
5. Implement creative ways to spark a conversation quickly.
6. Comprehensively assess participants’ social media imprint and presence and provide digital HIV care navigation using all the platforms participants actively use.
7. Use direct, succinct, but conversational language, especially with SMS text messaging and participants with limited literacy.
8. If your technology platform allows, use alternative media, such as GIFs, emojis, and memes, among others.
9. Use non-HIV-related messages to develop rapport and build trust.
10. Be responsive in real time, if at all possible. While SMS text messaging is asynchronous, make an effort to be quick to respond when participants choose to engage.
11. Carve out time to engage in a lengthier SMS text message session in real time. Designate a day and time in the week to conduct a quick text message chat.
12. Develop and integrate a feedback loop for digital HIV care navigation to inform primary care and the care team.
13. SMS text messaging may be a viable, sensitive method for evaluating when participants have fallen out of care and/or when a particular mode of communication (eg, phone number) is no longer viable.
14. Incorporate an assessment process to understand individuals’ attitudes toward digital technology; assess whether or not a digital intervention is suitable for a participant or not.
15. Do not use digital HIV care navigation to replace traditional navigation with high-acuity participants.

## Appendix

Multimedia Appendix 1 Peer-reviewer report from the Health Resources and Services Administration.

## Abbreviations

ART: antiretroviral therapy
CASI: computer-assisted self-interviewing
EMA: ecological momentary assessment
GED: General Educational Development
Health eNav: Health eNavigation
IRB: Institutional Review Board
MSM: men who have sex with men
PCMH: patient-centered medical home
SMS: short message service
YMSM: young men who have sex with men
