# Supplementary material for: Implementing a Digital HIV Care Navigation Intervention (Health eNav): Protocol for a Feasibility Study
Source: JMIR Res Protoc. 2019 Nov 8;8(11):e16406. doi: 10.2196/16406 (PMC6874804; doi:10.2196/16406)
Supplement: Multimedia Appendix 1 [file resprot_v8i11e16406_app1.pdf]

Health Resources and Services Administration
HRSA-15-029

HAB: Use of Social Media along the HIV Care Continuum - Demonstration Sites

Objective Review Committee Final Summary Statement

Score: 94

Application Number: 128516
Application Name: PUBLIC HEALTH FOUNDATION ENTERPRISES, INC.
State: CA City: City of Industry, CA

Criterion 1: NEED

Strength:

The applicant organization provides a comprehensive overview of the proposed needs assessment, detailing the extent of how the targeted population is reached.

The applicant organization clearly shows an understanding of the specific challenges within their community. For example, while the community is doing well getting People Living with HIV (PLWH) within the target population linked to care that there are shortcomings with regard to retention and viral suppression.

The applicant organization clearly demonstrates a thorough understanding of the unmet needs within the community through the sharing of surveillance data regarding the target population (including where they fall along the HIV care continuum).

Weakness:

None

Criterion 2: RESPONSE

Strength:

The applicant organization outlines a clear plan to enroll 325 participants in the target population over the project period.

The applicant organization outlines a strong plan for sustainability by gaining support of leadership within San Francisco's larger "Getting to Zero" initiative. After the project period, the applicant organization suggests including the proposed project into "Getting to Zero" while looking for additional funding.

The applicant organization identifies potential challenges and suggests suitable responses (i.e., integrating the proposed Digital Navigation project into LINCS for a smooth transition).

The application outlines a clear plan to include a "Digital Navigator" who will be available in real-time using social media (via text message, websites and geo-location strategies) in order to help move the target population along the HIV Care Continuum.

Weakness:

None

Criterion 3: EVALUATIVE MEASURES

Strength:

The applicant organization provides a comprehensive overview of the data management system.

Weakness:

None

Criterion 4: IMPACT

Strength:

The applicant organization describes their willingness to work with the Evaluation and Technical Assistance Center to ensure that the results of their project are disseminated widely including a report on the model and outcomes.

The applicant organization will submit presentations for consideration at scientific conferences.

Weakness:

None

Criterion 5: RESOURCES/CAPABILITIES

Strength:

The applicant organization details a plan to include staff (Principal Investigator and Evaluator) with the appropriate level of education and experience to successfully carry out all aspects of the project, including multi-site evaluations.

Weakness:

The application proposes hiring a "Digital Navigator" at the end of Year One of the project rather than earlier.

Criterion 6: SUPPORT REQUESTED

Strength:

The application meets the requirement for 0.25 FTE for Data Manager.

Weakness:

None
